# Effects of body mass index on the distributions, severity and surgical outcomes of chronic rhinosinusitis subtypes: a longitudinal study

**DOI:** 10.3389/falgy.2026.1808909

**Published:** 2026-05-29

**Authors:** Feng-Ling Yang, Biao Wang, Zhen-Hua Jiang, Li Xia, Wei Deng, Li-Jun Zhang, Shu-Hua Li, Yan Cai, Xiao-Xiang Li

**Affiliations:** 1 Department of Otorhinolaryngology-Head and Neck Surgery, Mianyang Central Hospital, Mianyang, China; 2 Department of Ophthalmology, The Third Hospital of Mianyang, Mianyang, China

**Keywords:** BMI, chronic rhinosinusitis, endoscopic sinus surgery, overweight or obese adults, propensity score weighted mixed effect model

## Abstract

**Objective:**

Metabolic disorders significantly affect patients’ physical health in various ways, yet their effects on different subtypes of chronic rhinosinusitis remain unclear. This study aimed to investigate how overweight and obesity affect the distributions, severity, and surgical outcomes of various chronic rhinosinusitis subtypes.

**Methods:**

Through a retrospective longitudinal analysis of the clinical data of 688 patients with chronic rhinosinusitis who underwent endoscopic sinus surgery in a tertiary hospital, the effects of BMI on chronic rhinosinusitis were evaluated.

**Results:**

The study found that the proportion of fungal sinusitis was lower in overweight or obese patients (*P* = 0.041). Patients who were overweight or obese had higher preoperative CT Lund‒Mackay scores (*P* = 0.005), and a similar trend was observed in those with neutrophil-infiltrated chronic rhinosinusitis (*P* = 0.035). By employing a propensity score weighted mixed effect model, the endoscopic scores of chronic rhinosinusitis patients who were overweight or obese were consistently higher than those of patients without these conditions at 1, 3, and 6 months after endoscopic sinus surgery (*P* < 0.001). This trend was observed both in patients with chronic rhinosinusitis with polyps and without polyps and in patients with eosinophil-infiltrated and neutrophil-infiltrated chronic rhinosinusitis.

**Conclusion:**

Overweight and obesity affect the distributions, severity, and surgical outcomes of different chronic rhinosinusitis subtypes. Therefore, the impact of BMI should also be considered when managing chronic rhinosinusitis.

## Introduction

Chronic rhinosinusitis (CRS) is characterized by persistent inflammation of the nasal cavity and sinus, which results from various factors. According to statistics, the incidence of CRS in China is approximately 8%, whereas in Europe and the United States, it exceeds 10% (1, 2). Despite having similar clinical manifestations, CRS is a highly heterogeneous disease. Several factors contribute to CRS, including anatomical abnormalities, ciliary dysfunction, allergies, microorganisms, bacterial superantigens, and rare conditions such as cystic fibrosis and nonsteroidal anti-inflammatory drug intolerance (3).

CRS varies by etiology, presenting different phenotypes and endotypes. Currently, CRS is classified into chronic sinusitis with nasal polyps (CRSwNP) and chronic sinusitis without nasal polyps (CRSsNP) according to clinical phenotypes. Based on endo-inflammation, CRS is currently subdivided into type 1, type 2 and type 3 inflammation. Type 2 CRS is often clinically characterized by nasal polyps and pathological eosinophil infiltration, with elevated IL-4, IL-5 and IL-13. INF-γ is the characteristic inflammatory factor of type 1 CRS, whereas type 3 CRS is characterized by elevated IL-17 and IL-22. In both type 1 and type 3 CRS, neutrophils are the main driving factors that disrupt the nasal mucosa barrier (2, 4).

The treatment approaches vary notably in accordance with CRS subtypes. In type 2 CRS, glucocorticoids are the cornerstone pharmacological treatment, yet fully controlling inflammation remains challenging. Current immunotherapies targeting IgE, IL-4, IL-5, and IL-13 are effective for refractory cases (5). In contrast, glucocorticoids are generally less effective in treating type 1 and type 3 inflammation, leading to different treatment strategies and outcomes for CRS (2).

Obesity is increasingly becoming a human health risk factor, affecting 40% of Americans and 8.1% of Chinese individuals (6, 7). Overweight and obesity, as measured by body mass index (BMI), can cause metabolic disorders and chronic inflammation, increasing the occurrence of diabetes, hypertension, and hyperlipidemia. In addition, overweight and obesity have been reported to increase the prevalence of allergic diseases such as asthma (8). Asthma, an allergic disease of the lower airway, is a representative inflammatory disease of the respiratory tract. Research indicates that obesity elevates leptin, adiponectin, INF-γ, IL-6, and IL-12 secretion in patients with asthma (9). It also skews CD4+ T cells to Th1-cell activation, which leads to worsened and uncontrollable type 1 inflammatory asthma, with ineffective glucocorticoid treatment (10, 11). Approximately 67% of patients with CRSwNP, a common inflammatory disease of the respiratory tract, are reported to have asthma (12). However, it remains unclear whether overweight and obesity affect the distribution and development of CRS, and whether they affect the treatment efficacy of different CRS subtypes.

Therefore, by analyzing the relationship between BMI and CRS, the current research aims to explore whether overweight and obesity affect the distributions, severity, and surgical outcomes of different CRS subtypes.

## Methods

### Patients and settings

This research was conducted in a tertiary referral center, and approved by the Clinical Research Management Board and the Ethics Committee at Mianyang Central Hospital (approval number: S202403199–01), and all methods were performed in accordance with relevant guidelines and regulations. Due to retrospective nature of the study, informed consent was waived by the ethics committee. This study retrospectively included patients with CRS treated at the Department of Otolaryngology, Mianyang Central Hospital. These patients underwent endoscopic sinus surgery (ESS) between January 1st, 2021, and January 1st, 2024. The diagnostic criteria for CRS are based on the European Position Paper on Rhinosinusitis and Nasal Polyps published in 2020 (2). Patients underwent detailed medical history, physical exams, routine tests, sinus CT scans, and nasal endoscopies. Those with allergic symptoms were tested for IgE and allergens. The inclusion criteria were as follows: (1) met the CRS diagnosis; (2) had complete medical records available; and (3) had undergone ESS. The exclusion criteria were as follows: (1) suffering from uncontrolled systemic diseases, such as uncontrolled malignancies or uncontrolled infection; and (2) having severe and uncontrolled psychological disorders.

Preoperative data collection included the following: (1) general information (sex, age, weight, height, smoking and drinking history, history of hypertension, diabetes, hyperlipidemia and hyperuricemia, allergy and asthma history); (2) laboratory examination data (blood eosinophils, basophils, blood glucose, uric acid, adenosine deaminase, cholinesterase, fucosidase, and urinary ketone); and (3) auxiliary examination data (Lund‒Mackay score of sinus CT and Lund‒Kennedy score of nasal endoscopy), both of which have been evaluated and recorded in medical records. As subjective assessment tools, the results were validated by reevaluating the CT and nasal endoscopy reports separately when data were collected by two otolaryngologists. If there were discrepancies, the two doctors discussed them until a final consensus was reached. (4) Pathological examination (presence of polyps and fungi and inflammatory cell infiltration in nasal-sinus samples) was performed.

This study categorized BMI into two groups according to Chinese BMI classification standards: the nonoverweight group (BMI < 24) and the overweight or obese group (BMI ≥ 24). Inflammatory cell infiltration was defined as follows: 10 or more eosinophils per high-power field for eosinophil-infiltrated CRS and 10 or more neutrophils per high-power field for neutrophil-infiltrated CRS.

All ESS procedures were conducted by the same surgical team with uniform postoperative follow-up protocols at 2 weeks, 1 month, 2 months, 3 months, 6 months, and 1 year postsurgery. At 1, 3, and 6 months post-ESS, nasal endoscopic Lund–Kennedy score evaluation was conducted.

### Statistical analysis

R statistical software (version 4.3.2; R Core Team, 2023) was used for description and data analysis. Independent sample *t*-tests, Wilcoxon rank sum tests, and $x^{2}$ tests were used to compare characteristics between different BMI groups and CRS subtypes. A propensity score weighted mixed effect model was applied to estimate the associations between BMI groups and repeatedly measured Lund–Kennedy scores. A *P* value lower than 0.05 was considered statistically significant.

## Results

### Characteristics and baseline data of the study subjects

**Figure 1:**
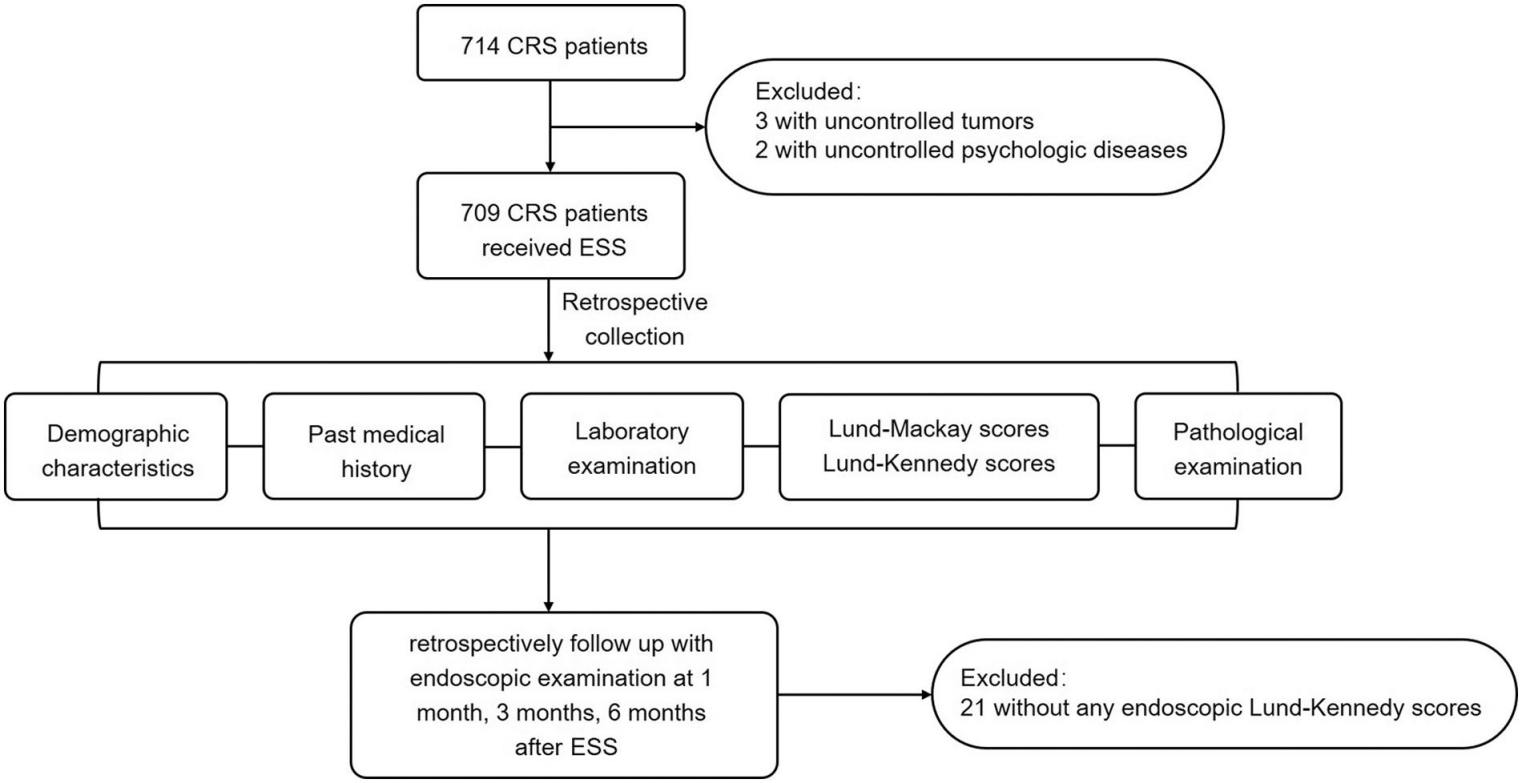
The participants enrollment and data collection of the study. CRS, chronic rhinosinusitis; ESS, Endoscopic sinus surgery.

The present study collected data from 714 CRS patients, while 21 patients failed to have any endoscopic Lund–Kennedy scores. Three patients with uncontrolled malignant tumors in other systems and two with severe uncontrolled psychological disorders were not included. Ultimately, data from a total of 688 patients with CRS were included. The data collection process is illustrated in Figure 1, and the results are summarized in Table 1.

**Table 1:** Descriptions of characteristics of the included participants.

Variables	$N(\text{\%})/\bar{x}(s)/M(IQR)$
Age (years)	47.15 (15.63)
Sex	
Male	296 (43.0)
Female	392 (57.0)
Race	
Han	687 (99.9)
Minority	1 (0.1)
Height (cm)	162.75 (8.40)
Weight (kg)	62.91 (11.50)
BMI (kg/m^2^)	23.65 (3.29)
BMI classification	
<24 kg/m^2^	379 (55.2)
≥24 kg/m^2^	308 (44.8)
Smoking history	136 (19.8)
Drinking history	88 (12.8)
History of asthma	30 (4.4)
History of allergy	18 (2.6)
History of diabetes	35 (5.1)
History of hyperlipidemia	3 (0.4)
History of hyperuricemia	2 (0.3)
History of hypertension	100 (14.5)
Laboratory examination	
IgE	40.00 (112.50) IU/mL
Serum Allergens	76 (13.3)
Adenosine deaminase	9.40 (3.55) U/L
Cholinesterase	8,697.80 (1,700.15) U/L
Fucosidase	33.10 (8.91) U/L
Blood glucose	5.13 (0.83) mmol/L
Uric acid	343.59 (94.85) umol/L
Complement C1	210.68 (43.03) mg/L
Fibrinogen	3.12 (0.94) g/L
White blood cell count	6.20 (1.76) × 10^9^/L
Number of neutrophils	3.41 (1.67) × 10^9^/L
Number of eosinophils	0.14 (0.19) × 10^9^/L
Number of basophils	0.03 (0.03) × 10^9^/L
Pathologic results	
Fungus	207 (30.1)
Polyps	417 (60.6)
Inflammatory cells	
Eosinophils	218 (31.7)
Neutrophils	449 (65.3)
CT Lund‒Mackay scores	10.42 (5.95)
Endoscopic Lund−Kennedy scores	
Preoperative	8.00 (6.00)
1 Month after ESS	5.00 (3.00)
3 Months after ESS	2.00 (2.00)
6 Months after ESS	2.00 (1.00)

### BMI and distributions of different CRS subtypes

In the nonoverweight group, 127 patients (33.3%) with fungal sinusitis were recorded, whereas 80 patients (26.1%) were recorded in the overweight or obese group. A statistically significant difference was found in the fungal sinusitis proportion between the two groups (*P* = 0.041).

In the nonoverweight group, 220 individuals (57.7%) had CRSwNP, whereas 197 individuals (64.4%) had CRSwNP in the overweight or obese group, with no significant difference in the proportion of CRSwNP between the two groups (*P* = 0.076).

In the nonoverweight group, 118 patients (30.9%) presented with histopathologically eosinophil-infiltrated CRS, whereas 100 patients (32.7%) presented with eosinophil-infiltrated CRS in the overweight or obese group, with no significant difference in the proportions of different inflammatory cell infiltration CRSs between the two groups (*P* = 0.627). The results above are summarized in Table 2.

**Table 2:** Components of different subtypes of CRS with different BMIs.

Pathologic Characteristics	BMI < 24 kg/m^2^ *N* = 381^a^	BMI ≥ 24 kg/m^2^ *N* = 306^a^	$x^{2}$	*P* value
Fungus	127 (33.3)	80 (26.1)	4.1753	0.041
Polyps	220 (57.7)	197 (64.4)	3.1530	0.076
Inflammatory cells			0.23676	0.627
Eosinophils	118 (30.9)	100 (32.7)		
** **Neutrophils	252 (66.1)	197 (64.4)		

a *n* (%).

### BMI and severity of different subtypes of CRS

In the nonoverweight group, the CT Lund‒Mackay score of the CRS was 9.0 (5.0, 14.0), and the endoscopic Lund‒Kennedy score was 8.0 (6.0, 12.0). In the overweight or obese group, the CT Lund–Mackay score was 11.0 (6.0, 15.0), and the endoscopic Lund–Kennedy score was 8.0 (6.0, 12.0). As detailed in Supplementary S1, a statistically significant difference in CT scores was detected between the two groups (*z* = −2.778, *P* = 0.005), whereas no significant difference in endoscopic scores was detected (*z* = −0.777, *P* = 0.437).

In the neutrophil-infiltrated CRS group, the CT Lund–Mackay score in the nonoverweight group was 8.19 ± 5.04, whereas that in the overweight or obese group was 9.22 ± 5.17. A significant difference in CT scores was found between the two groups (*P* = 0.035). However, both BMI groups had the same endoscopic Lund‒Kennedy score of 8.0 (6.0, 12.0).

### BMI and surgical outcomes of patients with CRS

Absolute correlation coefficients before and after propensity weighting were shown in Supplementary S2. After weighting, the average absolute value of the standardized mean differences between groups is less than 0.001, indicating that the covariates have reached a balanced state. For the interaction terms between BMI groups and follow-up times, three interaction terms, BMI≥24 kg/m^2^: postsurgery 1 Month; BMI ≥ 24 kg/m^2^: postsurgery 3 Months; and BMI ≥ 24 kg/m^2^: postsurgery 6 Months, were significant. The interaction term for BMI ≥ 24 kg/m²: Postsurgery 1 month was 1.007 (*P* < 0.001), indicating that patients with BMI ≥ 24 kg/m^2^ had an average endoscopic score 1.007 points higher than those with BMI < 24 kg/m² at one month postsurgery. The interaction term for BMI ≥ 24 kg/m²: Postsurgery 3 months was 0.893 (*P* < 0.001), indicating that patients with BMI ≥ 24 kg/m^2^ had an average endoscopic score 0.893 points higher than those with BMI < 24 kg/m² at three months postsurgery. The interaction term for BMI ≥ 24 kg/m²: Postsurgery 6 months was 0.753 (*P* < 0.001), indicating that patients with BMI ≥ 24 kg/m^2^ had an average endoscopic score 0.753 points higher than those with BMI < 24 kg/m² at six months postsurgery. These findings are summarized in Table 3.

**Table 3:** Propensity score weighted mixed effect model for association between BMI groups and repeated measured endoscopic scores of CRS after ESS.

Variables	B	95% CI	*P* value
BMI ≥ 24 kg/m^2^	−0.225	(−0.705, 0.255)	0.356
Time (Ref: pre-surgery)			
Time—Postsurgery 1 Month	−5.375	(−5.716, −5.034)	<0.001
Time—Postsurgery 3 Months	−8.354	(−8.695, −8.013)	<0.001
Time—Postsurgery 6 Months	−8.832	(−9.173, −8.491)	<0.001
Interaction: BMI ≥ 24 × Time			
BMI ≥ 24 kg/m^2^: postsurgery 1 Month	1.007	(0.508, 1.506)	<0.001
BMI ≥ 24 kg/m^2^: postsurgery 3 Months	0.893	(0.394, 1.392)	<0.001
BMI ≥ 24 kg/m^2^: postsurgery 6 Months	0.753	(0.254, 1.252)	<0.001

B, beta; CI, confidence interval.

### BMI and surgical outcomes of patients with different CRS subtypes

#### CRSwNP

By using a propensity score weighted mixed effect model, the interaction effects of BMI and follow-up time points in patients with CRSwNP were analyzed. One month postsurgery, the endoscopic score for CRSwNP patients with a BMI ≥ 24 kg/m² was 1.182 points higher than that for those with a BMI < 24 kg/m² (*P* < 0.001). At three months postsurgery, CRSwNP patients with a BMI ≥ 24 kg/m² had an endoscopic score 0.968 points higher than those with a BMI < 24 kg/m² (*P* = 0.002). At six months postsurgery, the endoscopic score for CRSwNP patients with a BMI ≥ 24 kg/m² was 0.819 points higher than that for those with a BMI < 24 kg/m² (*P* = 0.008). These findings are summarized in Table 4.

**Table 4:** Propensity score weighted mixed effect model for association between BMI groups and repeated measured endoscopic scores of CRSwNP after ESS and CRSsNP after ESS.

Variables	CRSwNP			CRSsNP		
	B	95% CI	*P* value	B	95% CI	*P* value
BMI ≥ 24 kg/m^2^	−0.410	(−0.997, 0.177)	0.171	−0.248	(−1.000, 0.503)	0.517
Time (Ref: pre-surgery)						
Time—Postsurgery 1 Month	−6.049	(−6.465, −5.632)	<0.001	−4.196	(−4.721, −3.671)	<0.001
Time—Postsurgery 3 Months	−9.160	(−9.577, −8.744)	<0.001	−6.816	(−7.341, −6.291)	<0.001
Time—Postsurgery 6 Months	−9.711	(−10.127, −9.295)	<0.001	−7.108	(−7.632, −6.583)	<0.001
Interaction: BMI ≥ 24 × Time						
BMI ≥ 24 kg/m^2^: postsurgery 1 Month	1.182	(0.574, 1.790)	<0.001	0.856	(0.086, 1.626)	0.029
BMI ≥ 24 kg/m^2^: postsurgery 3 Months	0.968	(0.360, 1.576)	0.002	0.919	(0.149, 1.689)	0.019
BMI ≥ 24 kg/m^2^: postsurgery 6 Months	0.819	(0.211, 1.427)	0.008	0.671	(−0.099, 1.440)	0.088

B, beta; CI, confidence Interval.

#### CRSsNP

By using a propensity score weighted mixed effect model, the interaction effects of BMI and follow-up time points in the CRSsNP were analyzed. One month postsurgery, the endoscopic score for CRSsNP patients with a BMI ≥ 24 kg/m² was 0.856 points higher than that for those with a BMI < 24 kg/m² (*P* = 0.029). At three months postsurgery, CRSsNP patients with a BMI ≥ 24 kg/m² had an endoscopic score 0.919 points higher than those with a BMI < 24 kg/m² (*P* = 0.019). For BMI ≥ 24 kg/m²: Postsurgery at 6 months, B = 0.671, there was no significant difference (*P* = 0.088). These findings are summarized in Table 4.

#### CRS infiltrated with eosinophils

By using a propensity score weighted mixed effect model, the interaction effects of BMI and follow-up time points in eosinophil-infiltrated CRS subtypes were analyzed. BMI ≥ 24 kg/m²: Postsurgery 1 month, *B* = 1.456, *P* = 0.001, indicating that patients with a BMI ≥ 24 kg/m² had an endoscopic score 1.456 points higher than those with a BMI < 24 kg/m² at one month postsurgery. For BMI ≥ 24 kg/m²: 3 months postsurgery, *B* = 0.885, *P* = 0.040, indicating that patients with BMI ≥ 24 kg/m² had an endoscopic score 0.885 points higher than those with BMI < 24 kg/m² at three months postsurgery. At six months postsurgery, for patients with a BMI ≥ 24 kg/m² (*B* = 0.640), no significant difference was observed (*P* = 0.137). These findings are summarized in Table 5.

**Table 5:** Propensity score weighted mixed effect model for association between BMI groups and repeated measured endoscopic scores of eosinophil-infiltrated CRS after ESS and neutrophil-infiltrated CRS after ESS.

Variables	Eosinophil-infiltrated CRS			Neutrophil-infiltrated CRS		
	B	95% CI	*P* value	B	95% CI	*P* value
BMI ≥ 24 kg/m^2^	−0.322	(−1.124, 0.479)	0.517	−0.220	(−0.811, 0.370)	0.465
Time (Ref: pre-surgery)						
Time—Postsurgery 1 Month	−6.678	(−7.235, −6.121)	<0.001	−4.755	(−5.170, −4.340)	<0.001
Time—Postsurgery 3 Months	−9.642	(−10.199, −9.084)	<0.001	−7.694	(−8.109, −7.279)	<0.001
Time—Postsurgery 6 Months	−10.113	(−10.670, −9.555)	<0.001	−8.154	(−8.569, −7.739)	<0.001
Interaction: BMI ≥ 24 × Time						
BMI ≥ 24 kg/m^2^: postsurgery 1 Month	1.456	(0.491, 2.421)	0.001	0.788	(0.191, 1.385)	0.01
BMI ≥ 24 kg/m^2^: postsurgery 3 Months	0.885	(−0.080, 1.849)	0.040	0.867	(0.270, 1.464)	0.005
BMI ≥ 24 kg/m^2^: postsurgery 6 Months	0.640	(−0.325, 1.604)	0.137	0.712	(0.116, 1.309)	0.02

B: beta; CI: confidence interval.

#### CRS infiltrated with neutrophils

By using a propensity score weighted mixed effect model, the interaction effects of BMI and follow-up time points in neutrophil-infiltrated CRS subtypes were analyzed. At one month post-ESS, the endoscopic score for patients with a BMI ≥ 24 kg/m² was 0.788 points higher than that for those with a BMI < 24 kg/m² (*P* = 0.01). At three months post-ESS, the endoscopic score for patients with a BMI ≥ 24 kg/m² was 0.867 points higher than that for those with a BMI < 24 kg/m² (*P* = 0.005). At six months post-ESS, the endoscopic score for patients with a BMI ≥ 24 kg/m² was 0.712 points higher than that for those with a BMI < 24 kg/m² (*P* = 0.02). These findings are summarized in Table 5.

## Discussion

This retrospective cohort study revealed that BMI influences the distribution, severity, and surgical outcomes of various subtypes of CRS. Among patients who required surgery, those who were overweight or obese had fewer patients with fungal sinusitis but more patients with CRSwNP or eosinophil-infiltrated CRS. Furthermore, CRS in patients who are overweight or obese is more severe. After ESS, patients who were overweight or obese had worse postsurgery recovery than those without these conditions.

Currently, the relationship between metabolic abnormalities and sinus inflammation has not been fully elucidated. Epidemiological studies have shown that a high BMI increases the risk of developing CRS (13, 14). Ulrika et al. (15) conducted a five-year prospective cohort study and reported that BMI is a long-term risk factor for CRS onset. Additionally, some studies categorized CRS into different subtypes to investigate how BMI affects the incidence of CRSwNP (16). These studies have focused on how BMI influences CRS incidence in the general population.

The present study examined how BMI influences CRS subtype distributions in patients requiring surgery and reported lower proportions of fungal sinusitis in overweight or obese individuals. Additionally, while not statistically significant, overweight or obese patients tended to have higher rates of CRSwNP and eosinophil-infiltrated CRS. This finding tended to be consistent with previous findings that high BMI increases the occurrence of CRSwNP (17). In overweight or obese individuals, the secretion of adipokines is altered, with decreased levels of anti-inflammatory factors such as lipocalin and increased levels of proinflammatory factors such as TNF-α, IL-6, and leptin (18, 19). Leptin, which is produced by adipose tissue and is used to regulate the neuroendocrine system and energy balance, is elevated in patients with eosinophil-infiltrated CRS and asthma. The leptin receptor in nasal polyp specimens also has increased expression (20–22). These phenomena indicate that leptin may mediate or exacerbate the occurrence of eosinophil inflammation locally or systematically. The fungal cases in our cohort were predominantly fungus balls, which are reported to be characterized by a non-eosinophilic profile, a milieu discordant with the eosinophil-promoting state of obesity, may partly explain the lower proportion of fungal cases in overweight/obese individuals (23). However, further investigation is still needed to clarify how BMI affects leptin and its impact on nasal inflammation.

Further analysis revealed that overweight or obese patients with neutrophil-infiltrated CRS had higher CT Lund–Mackay scores, indicating more severe inflammation. Previous studies have indicated that metabolic factors, such as hyperglycemia, can impair normal neutrophil functions, affecting their chemotaxis, adhesion, phagocytosis, and intracellular killing abilities. Therefore, the results of this study can probably be explained by the disruption of neutrophil functions due to metabolic issues related to overweight or obesity leading to more severe neutrophil-infiltrated sinusitis inflammation (24, 25). Notably, in CRS subtypes such as CRSwNP and CRSsNP, no significant difference in sinusitis severity with varying BMIs was observed. This may be due to discrepancies between the clinical and histopathological classifications. The clinical subtypes of CRSwNP include multiple pathological subtypes, leading to confounding results and elusive differences. Furthermore, no significant association was observed between BMI and disease severity in patients with eosinophil-infiltrated CRS. This is likely because this subtype is characterized by a generally high baseline severity, reflected in higher CT and endoscopic scores, which may mask the more subtle modulating effect of BMI.

This study revealed no significant differences in the endoscopic scores for various CRS subtypes across the different BMI groups before surgery. The endoscopic Lund‒Kennedy evaluation tool assesses nasal polyps, edema, rhinorrhea, scars, and crusts, with a primary focus on nasal cavity conditions. It cannot fully reflect sinus inflammation. Additionally, indicators such as scars and crusts focus on postsurgery recovery but not presurgery recovery.

Some studies have investigated how BMI influences postoperative recovery in CRS patients, which is closely linked to sinusitis recurrence and overall quality of life. A study involving 529 CRS patients revealed that those with both CRSwNP and metabolic syndrome had a greater likelihood of experiencing recurrence within two years of follow-up (26). The present study evaluated nasal endoscopic scores before and after surgery to examine the effect of BMI on short-term surgical recovery. Dynamic and continuous observation revealed higher endoscopic scores in the overweight or obese group at 1, 3, and 6 months postsurgery, indicating that worsening recovery conditions continued from 1 month to 6 months postsurgery in patients who were overweight or obese. This difference persisted in different subtypes of CRS, including CRSwNP and CRSsNP, as well as eosinophil-infiltrated and neutrophil-infiltrated CRS. This phenomenon demonstrates that higher BMI negatively influences surgical outcomes in various CRS subtypes, suggesting that obese patients are at greater risk of recurrence postsurgery.

This study employed the propensity score weighted mixed effect model method to analyze patient prognosis postsurgery. By utilizing propensity score weighting, this mixed-effects model controls for confounding factors and assesses the independent impact of BMI on recovery at different time points postsurgery. This approach minimizes biases and enhances the credibility of the findings (27, 28). This study included several factors in the model, including age, sex, history of hypertension and asthma, smoking history, laboratory indicators, and the presence of polyps. However, the psychological state and socioeconomic status of patients, lipid profiles and other metabolic biomarkers, as well as other combined treatments, such as glucocorticoids and nasal saline irrigation, may also affect the prognosis of ESS and should be considered. Future studies could incorporate these factors to provide a more thorough analysis of the surgical outcomes of CRS (29).

This study has certain limitations. To collect pathological data and subdivide CRSs on the basis of pathological findings, this study included only CRS patients who required surgery and underwent ESS in one surgical tertiary-care center. This may partly reflect the impact of BMI on the distributions of different CRS subtypes, but conclusions should be extrapolated to all CRS patients or the general population with caution. Confirmation in prospective, multicentre cohorts that include non-surgical patients is needed to validate and extend these findings. This study did not analyze several characteristic cytokines or adipokines associated with CRS, limiting the exploration of the potential mechanism by which BMI affects CRS. Future efforts should focus on validating our findings in multi-center cohorts and on comparing BMI on the immunology of different subtypes of CRS across different regions. With respect to the severity and surgical outcomes of CRS, it might be more comprehensive to include scales that evaluate subjective symptoms. Furthermore, the small size of the obese subgroup (BMI ≥ 28, *n* = 67) precluded its separate analysis, future large-scale studies are needed to define its specific association with CRS phenotypes.

## Conclusions

BMI, as a modifiable health measure, significantly affects the distributions, severity, and surgical outcomes of various subtypes of CRS. The present study highlights that for patients with CRS, controlling BMI and decreasing the risk of metabolic diseases can reduce disease severity and improve postoperative outcomes. Future in-depth research could explore the potential mechanisms by which overweight or obesity regulate various subtypes of CRS inflammation and their impacts on different subtypes of CRS from various aspects, such as clinical symptoms, signs, hematological and histopathological indicators.

## Data Availability

The raw data supporting the conclusions of this article will be made available by the authors, without undue reservation.
