# Supplementary material for: Effects of body mass index on the distributions, severity and surgical outcomes of chronic rhinosinusitis subtypes: a longitudinal study
Source: Front Allergy. 2026 May 29;7:1808909. doi: 10.3389/falgy.2026.1808909 (PMC13260235; doi:10.3389/falgy.2026.1808909)

**Supplementary 1 the CT and endoscopic scores of CRS with different BMIs**

| **Variables** | **BMI < 24 kg/m^2^**  (N = 381^1^) | **BMI ≥ 24 kg/m^2^**  (N = 306^1^) | $\boldsymbol{Z}$ | ***P* value** |
| --- | --- | --- | --- | --- |
| **CT scores** | 9.0 (5.0,14.0) | 11.0 (6.0,15.0) | -2.778 | 0.005 |
| **Endoscopic scores** | 8.0 (6.0,12.0) | 8.0 (6.0,12.0) | -0.777 | 0.437 |

^1^Median (IQR)

**Supplementary 2 Absolute correlation coefficients before and after propensity weighting**


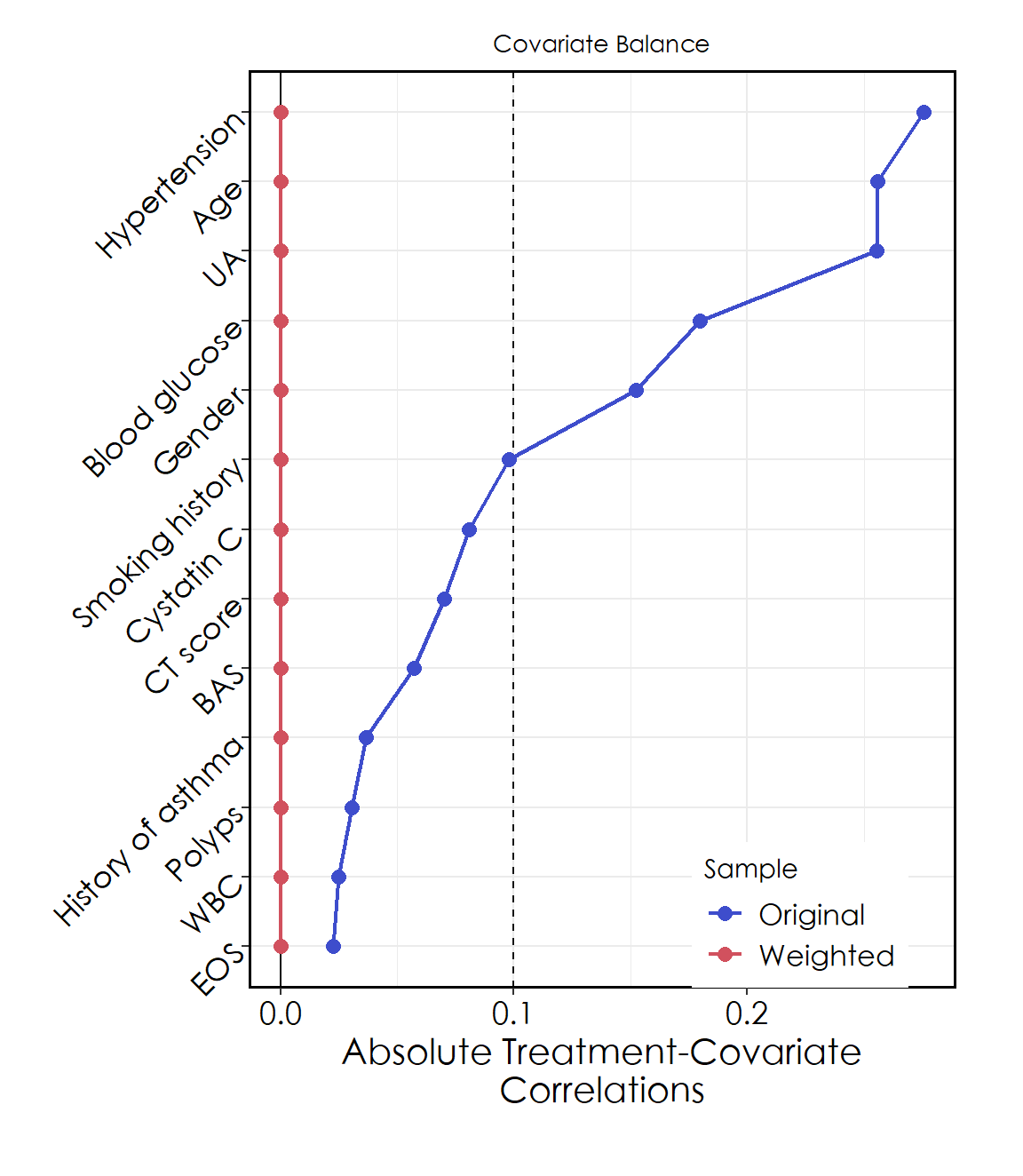

Supplement: Supplementary file 1 [file Table1.docx]
